# Kaempferol and Its Glycoside Derivatives as Modulators of Etoposide Activity in HL-60 Cells

**DOI:** 10.3390/ijms22073520

**Published:** 2021-03-29

**Authors:** Magdalena Kluska, Michał Juszczak, Jerzy Żuchowski, Anna Stochmal, Katarzyna Woźniak

**Affiliations:** 1 Department of Molecular Genetics, Faculty of Biology and Environmental Protection, University of Lodz, 90-236 Lodz, Poland; magdalena.kluska@edu.uni.lodz.pl (M.K.); michal.juszczak@edu.uni.lodz.pl (M.J.); 2 Department of Biochemistry and Crop Quality, Institute of Soil Science and Plant Cultivation, State Research Institute, 24-100 Pulawy, Poland; jzuchowski@iung.pulawy.pl (J.Ż.); asf@iung.pulawy.pl (A.S.)

**Keywords:** kaempferol, etoposide, DNA damage, apoptosis, cell cycle, oxidative stress, HL-60 cells

## Abstract

Kaempferol is a polyphenol found in a variety of plants. Kaempferol exerts antitumor properties by affecting proliferation and apoptosis of cancer cells. We investigated whether kaempferol and its glycoside derivatives—kaempferol 3-O-[(6-O-E-caffeoyl)-β-D-glucopyranosyl-(1→2)]-β-D-galactopyranoside-7-O-β-D-glucuropyranoside (P2), kaempferol 3-O-[(6-O-E-p-coumaroyl)-β-D-glucopyranosyl-(1→2)]-β-D-galactopyranoside-7-O-β-D-glucuropyranoside (P5) and kaempferol 3-O-[(6-O-E-feruloyl)-β-D-glucopyranosyl-(1→2)]-β-D-galactopyranoside-7-O-β-D-glucuropyranoside (P7), isolated from aerial parts of *Lens culinaris* Medik.—affect the antitumor activity of etoposide in human promyelocytic leukemia (HL-60) cells. We analyzed the effect of kaempferol and its derivatives on cytotoxicity, DNA damage, apoptosis, cell cycle progression and free radicals induced by etoposide. We demonstrated that kaempferol increases the sensitivity of HL-60 cells to etoposide but does not affect apoptosis induced by this drug. Kaempferol also reduces the level of free radicals generated by etoposide. Unlike kaempferol, some of its derivatives reduce the apoptosis of HL-60 cells (P2 and P7) and increase the level of free radicals (P2 and P5) induced by etoposide. Our results indicate that kaempferol and its glycoside derivatives can modulate the activity of etoposide in HL-60 cells and affect its antitumor efficacy in this way. Kaempferol derivatives may have the opposite effect on the action of etoposide in HL-60 cells compared to kaempferol.

## 1. Introduction

**Figure 1:**
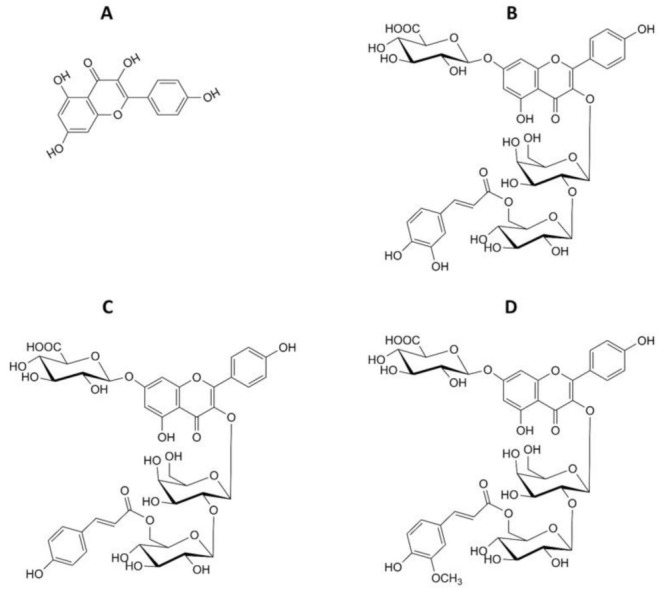
Chemical structures of kaempferol and its derivatives isolated from *Lens culinaris* Medik. (**A**) Kaempferol; (**B**) kaempferol 3-O-[(6-O-E-caffeoyl)-β-D-glucopyranosyl-(1→2)]-β-D-galactopyranoside-7-O-β-D-glucuropyranoside (P2); (**C**) kaempferol 3-O-[(6-O-E-p-coumaroyl)-β-D-glucopyranosyl-(1→2)]-β-D-galactopyranoside-7-O-β-D-glucuropyranoside (P5); (**D**) kaempferol 3-O-[(6-O-E-feruloyl)-β-D-glucopyranosyl-(1→2)]-β-D-galactopyranoside-7-O-β-D-glucuropyranoside (P7).

Kaempferol [3,5,7-trihydroxy-2-(4-hydroxyphenyl)-4H-1-benzopyran-4-one] (Figure 1A) is a flavonoid with an anticancer potential that can be found in a variety of plants and plant-derived food products, such as lentils, tea, broccoli or apples [1,2,3,4]. This flavonoid can modulate a number of key elements in cellular signal transduction pathways linked to apoptosis, angiogenesis, inflammation and metastasis [1]. Moreover, kaempferol, akin to other polyphenols, has strong antioxidant properties and can protect cells from oxidative stress. Oxidative stress plays a major role in the development of many diseases, including cancer. It is suggested that the anticancer properties of phenolic compounds such as kaempferol are in part due to their antioxidant properties. Both kaempferol and its glycoside derivatives show antioxidant activity by scavenging free radicals as well as inhibiting pro-oxidant enzymes and activating antioxidant enzymes [3]. Many studies have shown that polyphenols, including flavonoids, can sensitize cancer cells to anticancer drugs and radiotherapy, increasing their effectiveness [5,6]. The well-known examples in this respect are resveratrol [7,8], genistein [9], curcumin [10,11,12] and quercetin [13].

Chemotherapeutic agents used in leukemia treatment include topoisomerase II inhibitors, such as doxorubicin and etoposide. Etoposide (epipodophyllotoxin) is a semisynthetic derivative of podophyllotoxin, a nonalkaloid lignan isolated from dried roots and rhizomes of *Podophyllum peltatum* or *Podophyllum emodi (Berberidaceae)* [14]. A primary target for etoposide is topoisomerase II. Etoposide destroys cells by stabilizing a covalent enzyme-cleaved DNA complex, which is a transient intermediate in the catalytic cycle of topoisomerase II. The accumulation of cleavage complexes in treated cells leads to the generation of permanent DNA strand breaks, which can trigger cell deaths. The efficiency of etoposide’s cytostatic activity is dependent on the phase of the cell cycle, being the highest in the S phase [14]. Etoposide is also metabolized by cytochrome P450, horseradish peroxidase, and tyrosinase to an etoposide phenoxy radical, o-quinone-etoposide. The presence of the 4′-OH in etoposide has been found to be essential for the formation of the radical of this drug, its metabolites, as well as the antitumor activity [15]. Unfortunately, standard chemotherapy is nonspecific and affects both cancer and normal cells inducing a lot of side effects such as neutropenia, anemia, hair loss, and nausea. Therefore, we are looking for a compound which, on one hand, would enhance the effect of chemotherapeutic agents on cancer cells, and, on the other hand, protect normal cells. Such compounds may be the polyphenols. Recently, it has been demonstrated that kaempferol protected the vascular endothelium against doxorubicin-induced damage by regulating 14-3-3γ and ADMA/DDAHⅡ/eNOS/NO pathways, inhibiting oxidative stress, and improving mitochondrial function [16]. Other polyphenols, such as curcumin [17] and quercetin [18], also protect cardiomyocytes against doxorubicin-induced toxicity by suppressing oxidative stress and preventing mitochondrial dysfunction mediated by 14-3-3γ. We have previously observed that kaempferol glycosides isolated from aerial parts of *Lens culinaris* Medik.—kaempferol-3-O-[(6-O-E-feruloyl)-β-D-glucopyranosyl-(1→2)]-β-D-galactopyranoside-7-O-β-D-glucuro-pyranoside, kaempferol-3-O-{[(6-O-E-p-coumaroyl)-β-Dglucopyranosyl-(1→2)]-α-L-rhamnopyranosyl(1→6)}-β-D-galactopyranoside-7-O-α-L-rhamnopyranoside and kaempferol-3-O-[(6-O-E-caffeoyl)-β-D-glucopyranosyl-(1→2)]-β-D-galactopyranoside-7-O-(2-O-E-caffeoyl’)-β-D-glucuropyranoside—reduced DNA damage induced by etoposide in peripheral blood mononuclear cells (PBMCs), but did not have an impact on DNA damage in human promyelocytic leukemia (HL-60) cells [19].

Our earlier studies prompted us to the present examination of the effect of kaempferol and its glycoside derivatives (Figure 1) isolated from lentils on anticancer activity of etoposide in HL-60 cells. Here, we studied the effect of these plant compounds on cytotoxicity, apoptosis and reactive oxygen species (ROS) induced by etoposide. Furthermore, we tested the effect of kaempferol and its glycosides on DNA damage and cell cycle progression in HL-60 cells incubated with etoposide.

## 2. Results and Discussion

Different polyphenols, including kaempferol, have anticancer properties and can be used in cancer prevention and treatment. Flavonoids are found in plant material mainly in the form of glycosides [20]. Therefore, we decided to investigate the effects of not only kaempferol but also its glycoside derivatives isolated from aerial parts of *Lens culinaris* Medik. on the anticancer activity of etoposide in HL-60 cells. This is the first study that focuses on the effect of kaempferol and its glycosides on etoposide activity such as cytotoxicity, induction of apoptosis and DNA damage, cell cycle arrest and production of free radicals.

### 2.1. Cytotoxicity

**Figure 2:**
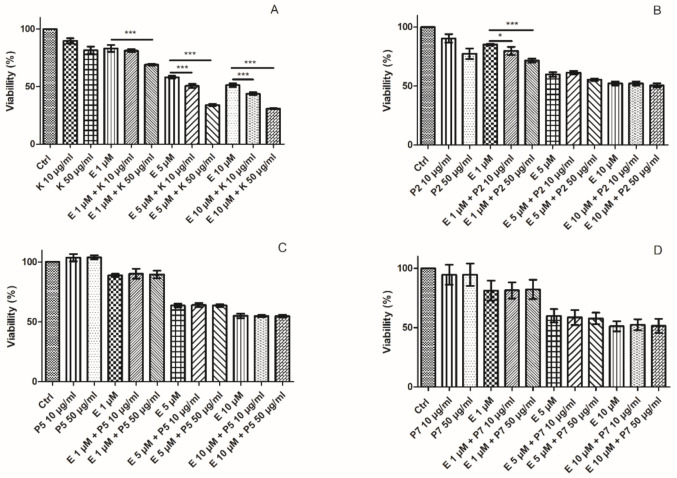
Viability of HL-60 cells determined by resazurin reduction assay after 24 h treatment with 1–10 µM etoposide (E) and 10-50 µg/mL kaempferol (K) (**A**), P2 (**B**), P5 (**C**) and P7 (**D**) derivatives. The figure shows mean results ± SD, *n* = 6; * *p* < 0.05, *** *p* < 0.001.

We observed that etoposide decreased the viability of HL-60 cells after 24 h incubation at all three tested concentrations (1, 5 and 10 µM) (Figure 2A). We also observed that coincubation of HL-60 cells with kaempferol and etoposide increased the cytotoxic effect of the drug notably. The incubation of HL-60 cells with 1 µM etoposide and 50 µg/mL kaempferol was more cytotoxic (69% of cell viability) than the incubation with only etoposide (83.2%) (*p* < 0.001) (Figure 2A). Moreover, kaempferol with 5 µM etoposide decreased cell viability from 58.2% to 50.6% and to 34% at 10 and 50 µg/mL concentrations, respectively (*p* < 0.001). In the case of 10 µM etoposide, coincubation with kaempferol at 10 and 50 µg/mL concentrations decreased cell viability from 51.2% to 43.8% and to 30.8%, respectively (*p* < 0.001). Next, we investigated the impact of kaempferol and its glycosides (P2, P5, P7), isolated from aerial parts of *Lens culinaris* Medik. on the cytotoxic effects of etoposide (Figure 2B,D). Kaempferol glycosides did not affect the cytotoxicity induced by etoposide in HL-60 cells. Only the P2 derivative decreased cell viability after incubation with 1 µM etoposide from 85.2% to 79.8% (*p* < 0.05) and 71.6% (*p* < 0.001) at 10 and 50 µg/mL concentrations, respectively (Figure 2B).

Our research indicates that only kaempferol has the effect of increasing the cytotoxicity of etoposide in HL-60 cells. Its glycoside derivatives tested by us did not affect the cytotoxicity of etoposide, except the P2 derivative, which decreased the viability of HL-60 cells incubated with 1 µM etoposide. The results obtained by us are consistent with the previous results. Studies carried out on the colon cancer LS174 cell line compared the cytotoxic activity of kaempferol and its analogs—i.e., kaempferol 3-O-glucoside and kaempferol 3-O-rutinoside isolated from the Tunisian quince *Cydonia oblonga* Miller [21]. Kaempferol, at a concentration of 120 µM, induced more than 80% of growth inhibition after 72 h incubation, while this effect did not exceed 30% and 37% for kaempferol 3-O-glucoside and kaempferol 3-O-rutinoside, respectively. Based on in silico analysis, it was suggested that these differences in antitumor activity between kaempferol and its analogs were caused by the absence of glucosyl groups in a kaempferol molecule. Moreover, it was shown that kaempferol displayed the highest antiproliferation effect on the human hepatoma cell line HepG2 (IC_50_ = 30.92 μM), mouse colon cancer cell line CT26 (IC_50_ = 88.02 μM) and mouse melanoma cell line B16F1 (IC_50_ = 70.67 μM) compared to kaempferol-7-O-glucoside, kaempferol-3-O-rhamnoside and kaempferol-3-O-rutinoside (IC_50_ > 100 μM after 24 h incubation) [22].

**Figure 3:**
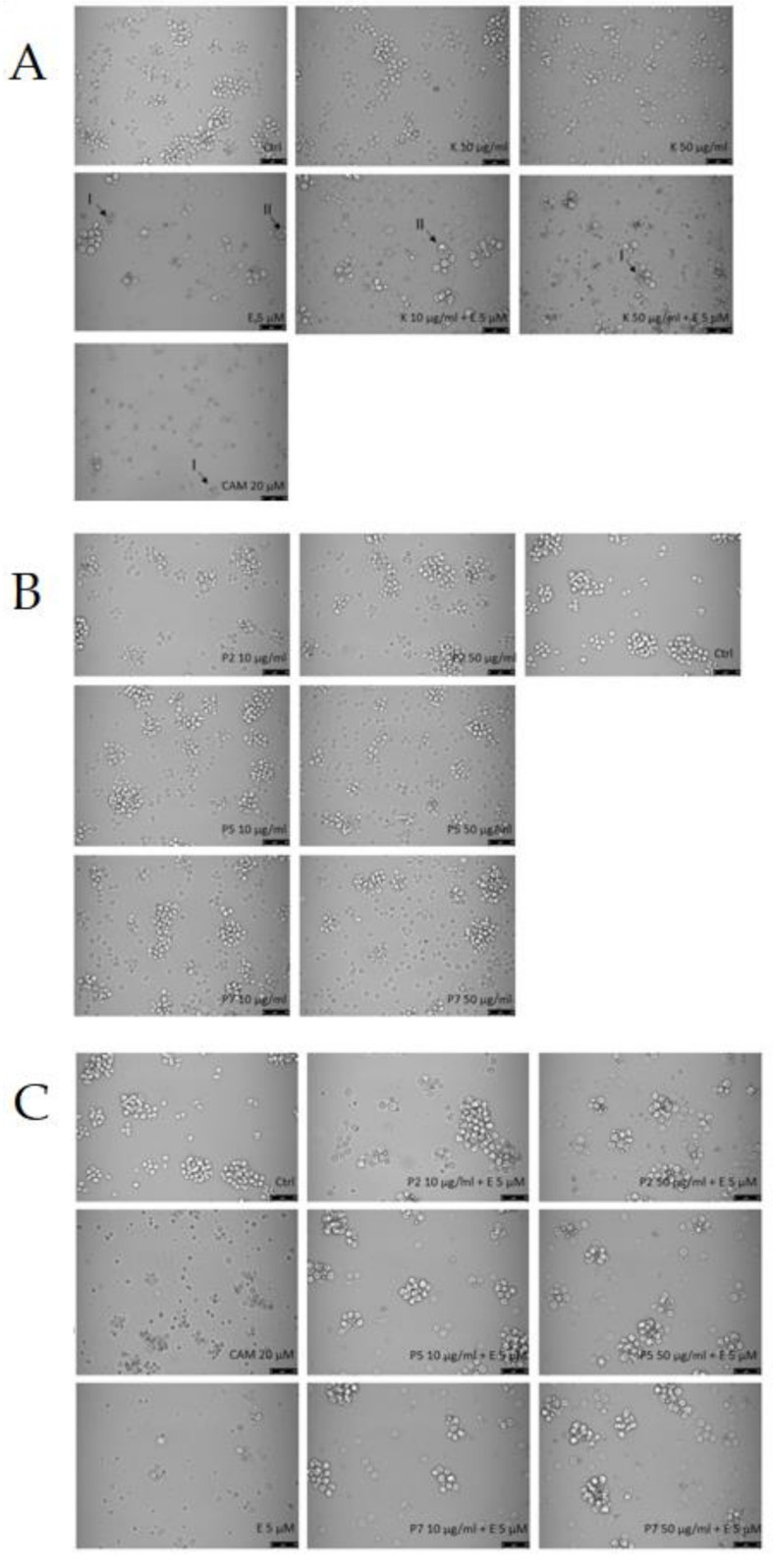
Morphological changes of human promyelocytic leukemia (HL-60) cells as examined by phase-contrast microscopy (magnification, ×200) after incubation with kaempferol and etoposide (**A**), kaempferol derivatives (**B**) or kaempferol derivatives and etoposide (**C**). Cells with fragmented nuclei (I) and enlarged cells (II).

Additionally, we examined the effect of etoposide, kaempferol and its glycoside derivatives on the morphology of HL-60 cells using phase-contrast microscopy (Figure 3A,C). Firstly, we observed that 5 µM etoposide decreased the number of HL-60 cells, with the cells becoming rough with shrinkage in comparison to the control cells. Moreover, the cells incubated with etoposide did not form clusters as control cells did (Figure 3A). Cells became enlarged and some of them had a fragmented nucleus. Similar changes in cell morphology were caused after incubation with 20 µM camptothecin (CAM), the inhibitor of topoisomerase I. Kaempferol also changed the morphology of cells, making them rough and not clustering as control cells did. Coincubation with etoposide and kaempferol led to a reduction in the number of cells and did not change their morphology. These results are consistent with our examination of HL-60 cells viability (Figure 2). We observed that kaempferol derivatives did not change the morphology of HL-60 cells (Figure 3B). Moreover, we detected that kaempferol derivatives protected HL-60 cells against morphological changes induced by etoposide (Figure 3C).

### 2.2. DNA Damage

**Figure 4:**
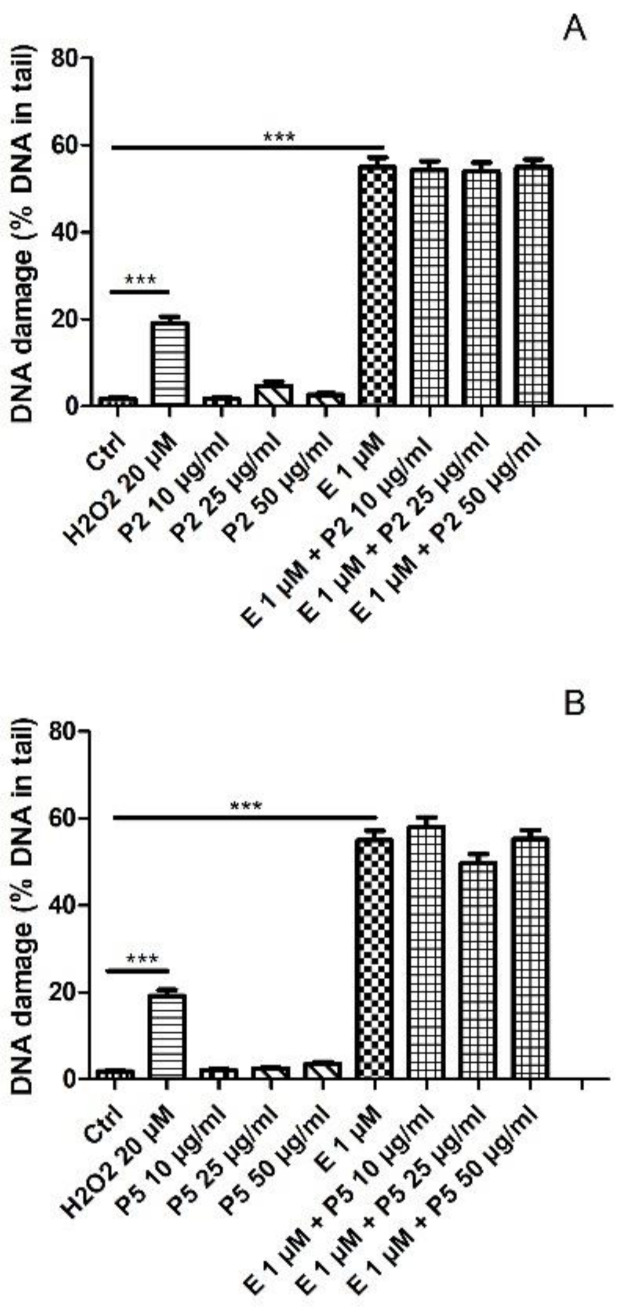
DNA damage, measured as the comet tail DNA (%) of HL-60 cells incubated for 2 h at 37 °C with P2 (**A**) and P5 (**B**) derivative (10–50 µg/mL) and 1 µM etoposide (E), analyzed by the alkaline comet assay. The figure shows mean results ± standard error of the mean (SEM), *n* = 100; *** *p* < 0.001.

**Figure 5:**
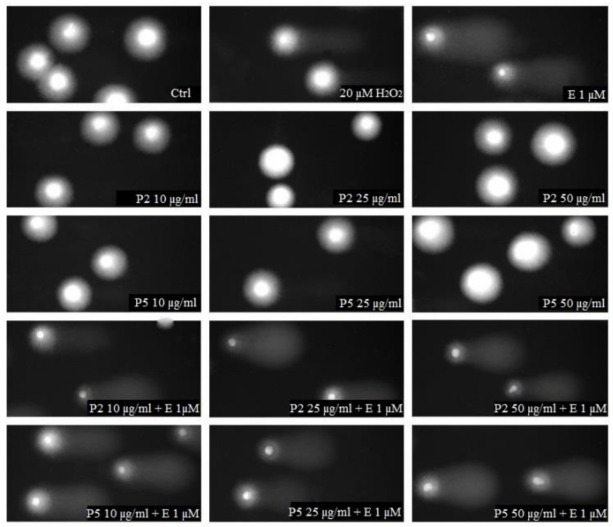
Representative pictures of comets obtained in the alkaline version of the comet assay after incubation of HL-60 cells with 10, 25 or 50 µg/mL P2 and P5 kaempferol derivatives and 1 µM etoposide (E). The figure also contains pictures of comets from negative control (Ctrl) and positive control (cells incubated with H_2_O_2_ at 20 µM for 15 min on ice).

Next, we tested the level of DNA damage following exposure to kaempferol derivatives and etoposide. Previously, we showed that kaempferol alone induced DNA damage in HL-60 cells as well as increased DNA damage induced by etoposide [19]. Coincubation of HL-60 cells with 1 µM etoposide and 50 μg/mL kaempferol increased the level of DNA damage from 27.5% to 39.6% (*p* < 0.001). Here, we did not observe any changes in the level of DNA damage induced by etoposide in HL-60 cells incubated with the two tested kaempferol derivatives (P2 and P5) (Figure 4). Figure 5 shows representative photos of the comets obtained after the incubation of HL-60 cells with these kaempferol derivatives (10–50 µg/mL) and 1 µM etoposide. The data are consistent with our previous results for two other kaempferol derivatives isolated from *Lens culinaris* Medik. [19].

Kaempferol is a well-known compound that induces apoptosis of cancer cells, one of the manifestations of which is DNA fragmentation [23,24,25]. Wu et al. (2015) observed when using the comet assay that kaempferol induced DNA damage in a dose-dependent manner in HL-60 cells [24]. Recent studies have also shown that bioflavonoids, including kaempferol, induce DNA double strand breaks (DSBs) and γH2AX foci formation [26]. Moreover, Western blotting indicated that kaempferol-decreased protein expression was associated with the DNA repair system, such as phosphate-ataxia-telangiectasia mutated (p-ATM), phosphate-ataxia-telangiectasia and Rad3-related (p-ATR), 14-3-3 proteins sigma (14-3-3σ), DNA-dependent serine/threonine protein kinase (DNA-PK), O^6^-methylguanine-DNA methyltransferase (MGMT), p53 and MDC1 protein expressions [24]. In mammalian cells, the phosphorylation of the subtype of histone H2A, called H2AX, in the position of Ser139 occurs in response to DSBs [27]. The phosphorylated form of H2AX is called γH2AX. Histone H2AX is a substrate for several phosphoinositide 3-kinase-related protein kinases (PIKKs), such as ATM, ATR or DNA-PK. Phosphorylation of H2AX plays a key role in DNA damage response (DDR) and is required for the assembly of DNA repair proteins at the sites containing damaged chromatin as well as for activation of checkpoints proteins which arrest the cell cycle progression [27].

### 2.3. Apoptosis

**Figure 6:**
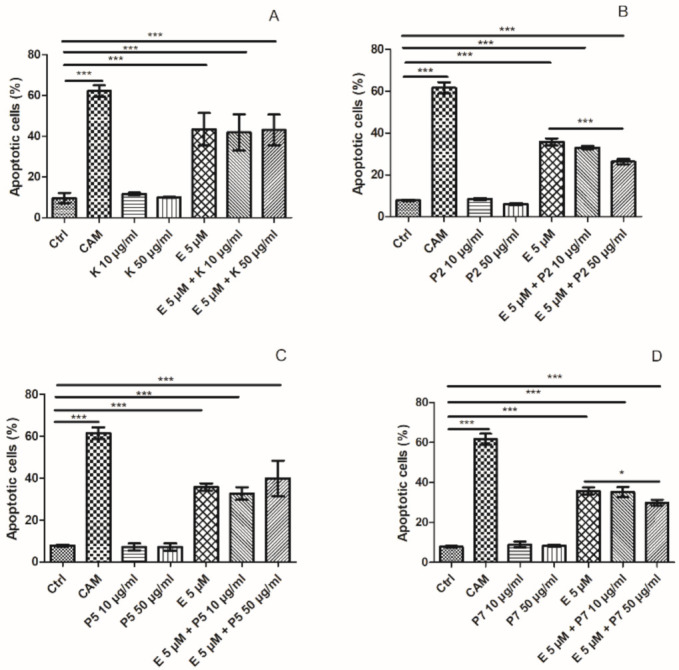
Apoptosis measured by flow cytometry using a double staining of FITC Annexin V and propidium iodide in HL-60 cells incubated for 24 h at 37 °C with 5 µM etoposide (E) and 10 or 50 µg/mL kaempferol (K) (**A**), P2 (**B**), P5 (**C**) and P7 (**D**) derivatives. The cells incubated with 20 µM camptothecin (CAM) for 24 h at 37 °C were positive control. The figure shows mean results ± SD, *n* = 3; * *p* < 0.05, 0.01, *** *p* < 0.001.

To investigate whether a drop in the viability of HL-60 cells, which we observed during the coincubation with kaempferol and etoposide, was due to an increase in programmed cell death, we examined the level of apoptotic HL-60 cells after incubation (24 h) with 5 µM etoposide, 10 and 50 µg/mL kaempferol, kaempferol derivatives (P2, P5, P7) and with combinations of these compounds. The cells incubated with 20 µM camptothecin (CAM) were the positive control [28]. Our results indicate that kaempferol does not have an impact on the level of apoptosis induced by etoposide in HL-60 cells (Figure 6A). Hence, an increase in the cytotoxic effect of etoposide after coincubation with kaempferol is not associated with apoptosis. Studies carried out on HL-60 cells demonstrated that quercetin protected cells from apoptosis induced by etoposide [29]. However, quercetin at low concentrations (5–30 µM) attenuated the therapeutic effects of cisplatin and other antineoplastic drugs in ovarian cancer cells by reducing ROS damage and increasing the expression of endogenous antioxidant enzymes, suggesting a ROS-mediated mechanism of attenuating anticancer drugs [30]. On the other hand, another polyphenol, curcumin, significantly enhanced apoptosis induced by etoposide in HL-60 cells [12].

Our result is somewhat surprising given that, as we have previously shown, kaempferol increased etoposide-induced DNA damage in HL-60 cells [19]. Moreover, kaempferol induces apoptosis of cancer cells which has been confirmed in numerous experiments. Moradzadeh et al. showed that kaempferol at the concentrations of 50 and 100 µM decreased the viability of HL-60 cells after 72 h incubation [31]. Incubation with kaempferol for 72 h enhanced apoptosis in HL-60 cells via both intrinsic and extrinsic pathways. We did not observe apoptosis after 24 h incubation of HL-60 cells with kaempferol (10 and 50 µg/mL) (Figure 6A). This is probably due to the shorter incubation time used by us compared to other studies [31]. However, we deliberately used a shorter incubation time with kaempferol to prevent cell apoptosis. In our opinion, only then can we evaluate the effect of kaempferol on etoposide-induced apoptosis. It was also shown that kaempferol decreased the viability and induced the apoptosis of HepG2 hepatocellular carcinoma cells [32]. Moreover, kaempferol also reduced the viability and increased the level of apoptosis in human cervical cancer (HeLa) cells [33]. These studies also showed that kaempferol had no toxic effect on normal human foreskin fibroblast (HFF) cells. Kaempferol derived from *Semecarpus anacardium* protected normal lung and liver cells from apoptosis induced by H_2_O_2_ [34]. This protective effect was associated with an increased expression of proteins responsible for the response to oxidative stress, such as Nrf2, superoxide dismutase (SOD), catalase and phospho-p38 MAPK.

We observed that all three tested kaempferol glycosides did not induce apoptosis in HL-60 cells by themselves (Figure 6B,D). Moreover, we noticed that the P2 derivative at the concentration of 50 µg/mL reduced the level of apoptosis induced by etoposide from 35.8% to 26.3% (*p* < 0.001) (Figure 6B). Similarly, the P7 derivative decreased the level of apoptotic cells induced by etoposide from 35.8% to 29.8% (*p* < 0.05) (Figure 6D). The opposite effect on apoptosis of leukemic cell lines U937, K562, and HL-60 cells was observed in the case of kaempferol-3-O-[α-l-rhamnopyranosyl-(1→4)-O-α-l-rhamnopyranosyl-(1→6)-O]-β-d-glucopyranoside isolated from *Wattakaka volubilis* [35]. This glycoside derivative exhibited antileukemic activity with IC_50_ values of 13.5, 10.8, and 13.2 μg/mL in U937, K562, and HL-60 cell lines, respectively. The flow-cytometric analysis confirmed that the cell cycle arrest occurred at G1 phase in the case of U937 and K562 cell lines and G2/M phase in the case of HL-60 cell lines. Moreover, both confocal microscopic analysis and DNA laddering assay confirmed the apoptosis and cell cycle arrests of leukemic cells [35].

Summarizing our results on the effect of kaempferol and its derivatives on etoposide-induced apoptosis, it should be stated that kaempferol, in addition to strong proapoptotic properties, also exhibits strong antioxidant properties that can protect cancer cells against apoptosis. Our results indicate that glycoside derivatives of kaempferol, such as the compounds P2 and P7, which we analyzed, may have stronger antiapoptotic properties than kaempferol.

### 2.4. Cell Cycle

Next, we investigated the cell cycle progression in HL-60 cells after 24 h incubation with etoposide, kaempferol and its glycoside derivatives (Table 1). We observed that both kaempferol and its glycoside derivatives did not alter the progression of cell cycle. We also observed an increase in cell level in sub-G1 phase from 1.1% to 10.5% after incubation with 0.5 µM etoposide (*p* < 0.01). Moreover, the G0/G1 fraction decreased significantly in response to 0.5 µM etoposide compared to the control cells from 42.4% to 20% (*p* < 0.001). The presence of cells in the sub-G1 phase is associated with apoptosis and DNA fragmentation. This result confirms the strong apoptotic effect of etoposide, as also shown in the experiment with double staining of cells with annexin V and propidium iodide (Figure 6). Our results are in agreement with the data obtained by Żuryń and colleagues on HL-60 cells treated with 0.5–1 µM etoposide [36]. We also observed a slight decrease in the number of S phase cells after incubation with etoposide (*p* < 0.05). Additionally, etoposide increased the population of cells in G2/M phase from 24.4% to 50.2% (*p* < 0.001). We noticed that both kaempferol and its glycoside derivatives had no impact on progression of cell cycle in cells incubated with etoposide (Table 1).

Results of transcriptomic analysis supported by real-time qRT-PCR, obtained with the human fibroblast model, indicate that polyphenols, including kaempferol, may regulate cell cycle and DNA replication in human cells due to modulation of expression of a relatively large group of genes whose products are involved in these processes [37]. For example, it was demonstrated that kaempferol induced G2/M cell cycle arrest via the Chk2/Cdc25C/Cdc2 pathway and Chk2/p21/Cdc2 pathway in human ovarian cancer A2780/CP70 cells [38]. Kaempferol-mediated antitumor activity toward Jurkat T cells was attributable to G2-checkpoint activation, which not only caused G2-arrest of the cell cycle, but also the phosphorylation of p53 (Ser-15) and subsequent induction of mitochondria-dependent apoptotic events [39].

Certain bioflavonoids such as kaempferol, quercetin and myricetin have been shown to directly inhibit topoisomerase II, similarly to etoposide [40]. Recent studies have shown that myricetin, genistein, and quercetin act most similarly to etoposide, although with varying topoisomerase II dependence. By contrast, kaempferol and luteolin have distinct kinetics that are mostly topoisomerase II-independent [26]. However, it should be emphasized that bioflavonoids can have a pleiotropic effect. Along with the ability to poison topoisomerase II, bioflavonoids can impact DNA repair processes, alter epigenetic markers, and activate signal transduction pathways, leading to altered protein expression in multiple pathways, such as cell cycle regulation, cell survival and cytokine expression [26,37]. The ability to inhibit topoisomerase II by substances of plant origins may lead to their antagonistic activity with etoposide in cancer cells [41].

### 2.5. Free Radicals

**Figure 7:**
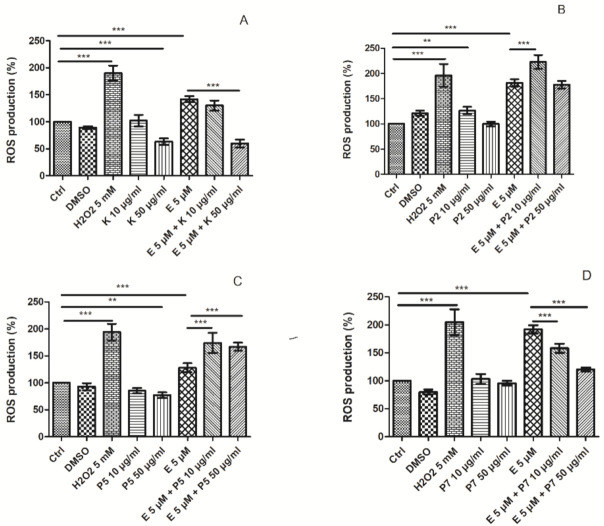
Reactive oxygen species (ROS) level in HL-60 cells incubated for 24 h at 37 °C with 5 µM etoposide (E) and 10 µg/mL or 50 µg/mL kaempferol (K) (**A**), P2 (**B**), P5 (**C**) and P7 (**D**) derivatives. The cells incubated with 5 mM H_2_O_2_ for 15 min at 37 °C were positive control. The figure shows mean results ± SD, *n* = 6; ** *p* < 0.01, *** *p* < 0.001.

**Figure 8:**
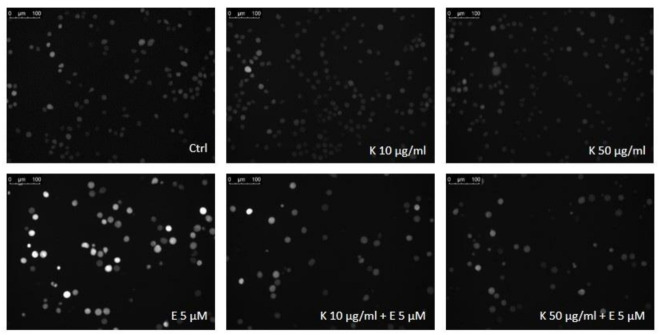
Representative photos of cells with 20 the fluorescence probe H_2_DCFH-DA after 24 h incubation at 37 °C with 5 µM etoposide (E) and 10 µg/mL or 50 µg/mL kaempferol (K). The figure also contains pictures of comets from negative control (Ctrl).

Firstly, we observed that 5 µM etoposide induced the statistically significant increase in the level of intracellular ROS (*p* < 0.001) (Figure 7 and Figure 8). This confirms that etoposide at this concentration induces oxidative stress in HL-60 cells. Next, we investigated the induction of intracellular ROS in the cells treated with 10 and50 µg/mL kaempferol or its glycoside derivatives (P2, P5, P7) and in combination with 5 µM etoposide. We observed that 50 µg/mL kaempferol decreased the level of intracellular ROS in HL-60 cells (*p* < 0.001) compared to the control cells, which indicates that kaempferol reduces endogenous free radicals (Figure 7A). Moreover, 50 µg/mL kaempferol decreased the level of ROS induced by 5 µM etoposide (*p* < 0.001) (Figure 7A and Figure 8). We did not observe any changes in ROS induction by etoposide in the case of 10 µg/mL kaempferol. The presented results showed that the enhancement of etoposide cytotoxicity by kaempferol was not due an increase in ROS generation. The antioxidant activity of kaempferol does not limit the activity of the drug. Our results confirm the previous studies which demonstrated the antioxidant properties of kaempferol [21,22,34]. Not only is kaempferol a potent scavenger of superoxide anion, hydroxyl radical and peroxynitrite, but it also inhibits pro-oxidant enzymes, such as xanthine oxidase, and activates antioxidant enzymes such as superoxide dismutase, catalase and heme oxygenase-1, and even prevents the generation of hydroxyl radicals by chelating cuprous or ferrous. Moreover, kaempferol contains hydroxyl groups at C3, C5, and C4, an oxo group at C4, and a double bond at C2-C3 that might explain its antioxidant activity [3]. Kaempferol can control the cancer through its antioxidative/antinitrosative potential by restoring cell redox hemostasis by inhibiting the NF-κB pathway and upregulating the Nrf2 transcriptional pathway [3,34].

Interestingly, we observed that the P2 derivative increased the level of intracellular ROS at the concentration of 10 µg/mL compared to the control cells (*p* < 0.01) (Figure 7B). Moreover, coincubation of 5 µM etoposide with 10 µg/mL P2 and P5 at 10 and 50 µg/mL elevated the ROS level in comparison to the incubation with etoposide only (*p* < 0.001) (Figure 7B,C). However, this growth in ROS generation did not increase the cytotoxicity of etoposide. Our results suggest that the glycoside derivatives of kaempferol may act differently compared to kaempferol. On the other hand, P5 at the concentration of 50 µg/mL decreased the ROS level to 77% compared to the control (*p* < 0.01) (Figure 7C). In contrast to P2 and P5, and similarly to kaempferol, the P7 derivative significantly decreased the level of ROS induced by etoposide (*p* < 0.001) (Figure 7D).

It was proposed that the synergistic enhancement of activity of anticancer drugs was caused by polyphenols: quercetin, apigenin, emodin, rhein and *cis*-stilbene are associated with the ability to change the glutathione (GSH) level in leukemic cells [42]. In the two lymphoid cell lines, CCRF-CEM and Jurkat, it was shown that all studied polyphenols when used in combination with topoisomerase II inhibitor, etoposide or doxorubicin, caused a synergistic or additive decrease in cell proliferation, G_2_M or S phase cell cycle arrest and apoptosis. This was associated with a synergistic/additive reduction in GSH levels, increased activity of caspases 3, 8 and 9 and DNA damage. Similar effects were observed in the myeloid cell lines, THP-1 and KG1a, when quercetin and apigenin were used in combination with topoisomerase II inhibitor. However, when emodin, rhein and to a lesser extent *cis*-stilbene were used in combination with etoposide or doxorubicin, there was an antagonistic increase in ATP, an inhibition of apoptosis and no cell cycle arrest. This was associated with an elevation of GSH levels and reduction in activity of caspases 3, 8 and 9, and little or no DNA damage [42].

## 3. Materials and Methods

### 3.1. Reagents

3,4′,5,7-Tetrahydroxyflavone (kaempferol) (K0133), 4′-demethylepipodophyllotoxin 9-(4,6-O-ethylidene-β-D-glucopyranoside) (etoposide) (E1383), low-melting-point (LMP) and normal-melting-point (NMP) agarose, 4′,6-diamidino-2-phenylindole (DAPI), dimethyl sulfoxide (DMSO), camptothecin (C9911), nocodazole (M1404), 2′,7′-dichlorofluorescein diacetate (H_2_DCFDA), resazurin sodium salt (R7017) and hydrogen peroxide (H_2_O_2_) were purchased from Sigma-Aldrich (St. Louis, MO, USA). Kaempferol was dissolved in DMSO and stored at −20 °C. Etoposide was dissolved in methanol.

### 3.2. Kaempferol Glycosides from the Aerial Parts of Lentil

Kaempferol glycosides: kaempferol 3-O-[(6-O-E-caffeoyl)-β-D-glucopyranosyl-(1→2)]-β-D-galactopyranoside-7-O-β-D-glucuropyranoside (P2), kaempferol 3-O-[(6-O-E-p-coumaroyl)-β-D-glucopyranosyl-(1→2)]-β-D-galactopyranoside-7-O-β-D-glucuropyranoside (P5) and kaempferol 3-O-[(6-O-E-feruloyl)-β-D-glucopyranosyl-(1→2)]-β-D-galactopyranoside-7-O-β-D-glucuropyranoside (P7) were isolated from aerial parts of *Lens culinaris* Medik. according to the procedure described by Żuchowski et al. (2014) [43]. All isolated kaempferol glycosides were dissolved in 50% DMSO and stored at −20 °C.

### 3.3. Cell Preparation

The human promyelocytic leukemia (HL-60) cell line was obtained from the American Type Culture Collection (ATCC). The cells were cultured in flasks at 37 °C in 5% CO_2_ atmosphere in Iscove’s Modified Dulbecco’s Medium (IMDM) with 2 mM L-glutamine, 25 mM HEPES (Lonza, Basel, Switzerland), 15% inactivated fetal bovine serum (FBS) and penicillin/streptomycin solution (100 U/mL and 100 μg/mL, respectively).

### 3.4. Cell Treatment

In all experiments, the HL-60 cells were seeded in the culture medium and then incubated at 37 °C with different concentrations (10–50 µg/mL) of kaempferol and kaempferol derivatives: (P2), (P5) and (P7). The cells were also treated with different concentrations of etoposide (1 or 5 µM) and incubated for 2 h at 37 °C until DNA damage induction or for 24 h at 37 °C to measure viability, apoptosis, cell cycle, ROS induction and gene expression. The final concentration of DMSO and methanol in the samples did not exceed 0.5% [19].

### 3.5. Cell Viability

Cell viability was measured using the resazurin reduction assay based on the ability of viable cells to reduce resazurin to fluorescent resorufin. The HL-60 cells were seeded on 96-well plates at a density of 1 × 10^4^ cells/mL in a culture medium and incubated for 24 h with the tested compounds (etoposide, kaempferol, P2, P5, P7) at 37 °C in 5% CO_2_. The assay was described in detail by Juszczak et al. (2020) [44].

### 3.6. DNA Damage

DNA damage was estimated using the alkaline comet assay according to the procedure defined by Singh et al. (1988) [45], which was described in detail in the authors’ previous work [19].

### 3.7. Apoptosis

Apoptosis was measured using the FITC Annexin V Apoptosis Detection Kit II (BD Biosciences, San Jose, USA). The HL-60 cells were seeded in 6-well plates at density of 2 × 10^5^ cells/mL. The cells incubated with 20 µM camptothecin (CAM) for 24 h at 37 °C were the positive control. After incubation, the cells were collected and washed twice with ice-cold PBS. The cells were resuspended in 1 × Binding Buffer (100 µL) and incubated with FITC Annexin V (5 µL) and propidium iodide (PI) (5 µL) for 15 min at room temperature in the dark. Then, 400 µL of Binding Buffer was added to each tube and the samples were measured within 1 h using the LSRII flow cytometer (Becton Dickinson, San Jose, leCA, USA) equipped with 488 nm laser excitation and BD FACSDiva software v 4.1.2. The percentage of apoptotic cells was expressed as a population of FITC Annexin V-positive cells [28].

### 3.8. Cell Cycle

The HL-60 cells were seeded in 6-well plates at density of 0.5 × 10^6^ cells/mL and incubated with the tested compounds. The cells incubated with 100 ng/mL nocodazol (NOC) for 24 h at 37 °C were the positive control. Next, the cells were collected and washed twice with PBS. Then, the cells were resuspended in PBS and allowed to cool for 15 min on ice. Then, one volume of −20 °C absolute ethanol was added, and the samples were stored at 4 °C. Before the analysis, samples were pelleted and resuspended in 300 µL of staining solution containing 40 µg/mL PI and 200 µg/mL RNase A. Samples were incubated for 30 min at 37 °C in the dark until analysis. DNA content was analyzed using the LSRII flow cytometer (Becton Dickinson, San Jose, CA, USA) [28].

### 3.9. Detection of Reactive Oxygen Species

The production of intracellular reactive oxygen species (ROS) was determined by measuring the fluorescence of 2′,7′-dichlorofluorescein diacetate (H_2_DCFDA). The cells (final density of 5 × 10^5^ cells/mL) were seeded in 6-well plates and incubated with 5 µM etoposide and 10 or 50 µg/mL kaempferol, P2, P5 and P7 derivatives for 24 h at 37 °C. The cells incubated for 15 min with 5 mM H_2_O_2_ at 37 °C were the positive control. The assay was described in detail by Juszczak et al. (2020) [44].

### 3.10. Statistical Analysis

The values of the comet assay were expressed as mean + standard error of the mean (SEM) from two experiments; data from the experiments were pooled and statistical parameters were calculated. The data from cell viability, apoptosis detection, cell cycle distribution and ROS measurement are presented as mean values ± SD from 3–6 independent experiments. Statistical analyses were performed using GraphPad Prism 5 (GraphPad Software Inc., La Jolla, CA, USA). Statistical differences were determined by means of a one-way ANOVA analysis followed by post hoc Tukey’s multiple comparison test. The differences were considered to be statistically significant when the *p* value was less than 0.05.

## 4. Conclusions

Our results suggest that kaempferol is a rather weak modulator of etoposide activity in HL-60 cells. Perhaps this is due to the compensation of the effects of kaempferol, its derivatives and etoposide. Kaempferol increases the sensitivity of HL-60 cells to etoposide but does not increase apoptosis and ROS level induced by etoposide. Our study also shows that glycoside kaempferol derivatives, isolated from *Lens culinaris* Medik., can limit the apoptotic activity of etoposide in cancer HL-60 cells. However, the results presented here concern HL-60 cells only, which is a major limitation of our work. It would be interesting to assess how kaempferol and its glycoside derivatives affect the activity of etoposide in other cancer cells. 

It seems that kaempferol and its glycoside derivatives may act similarly to emodin, rhein and *cis*-stilbene in combination with etoposide [42]. It cannot be ruled out that kaempferol and its derivatives, due to their strong antioxidant properties, will exert an antagonistic effect on the effect of etoposide action. Moreover, it is worth emphasizing that some of kaempferol derivatives may act differently than kaempferol. Our results indicate the need for further and more detailed research into the interactions between polyphenols, such as kaempferol and its derivatives, and chemotherapeutic agents used in the treatment of cancer.

## Figures and Tables

**Table 1:** Cell cycle distribution measured by flow cytometry using a staining with propidium iodide in HL-60 cells incubated for 24 h at 37 °C with 0.5 µM etoposide (E) and 10 or 50 µg/mL kaempferol (K), P2, P5 and P7 derivatives. The cells incubated with 100 ng/mL nocodazole (NOC) for 24 h at 37 °C were positive control.

Treatment/Concentration	DNA Content (%)			
	sub-G1	G0/G1	S	G2/M
Ctrl	1.1 ± 0.6	42.4 ± 3.4	32.1 ± 1.2	24.4 ± 1.9
NOC	23.8 ± 1.3 ***	33.7 ± 9.6	13.8 ± 5.7 ***	28.7 ± 7.1
K 10 µg/ml	1.2 ± 0.6	42.5 ± 3.0	29.0 ± 3.0	27.4 ± 1.1
K 50 µg/ml	1.5 ± 1.0	33.7 ± 5.7	36.4 ± 7.3	28.4 ± 3.1
P 2 10 µg/ml	1.1 ± 0.9	40.6 ± 6.5	31.6 ± 6.0	26.7 ± 1.7
P 2 50 µg/ml	1.1 ± 0.4	44.0 ± 4.1	28.7 ± 2.9	26.2 ± 1.8
P 5 10 µg/ml	1.0 ± 0.9	43.9 ± 1.8	27.8 ± 1.8	27.3 ± 2.6
P 5 50 µg/ml	1.2 ± 0.9	43.0 ± 5.8	32.3 ± 5.5	23.5 ± 0.5
P 7 10 µg/ml	1.1 ± 0.8	43.7 ± 6.4	30.2 ± 5.4	25.0 ± 1.6
P 7 50 µg/ml	1.2 ± 0.4	46.8 ± 2.2	28.6 ± 1.9	23.4 ± 0.5
E 0.5 µM	10.5 ± 6.0 **	20.0 ± 2.6 ***	19.3 ± 2.6 *	50.2 ± 6.0 ***
E 0.5 µM + K 50 µg/ml	11.0 ± 4.2 **	14.5 ± 1.3 ***	18.4 ± 0.6 *	56.1 ± 5.6 ***
E 0.5 µM + P 2 50 µg/ml	9.4 ± 3.1 *	14.3 ± 2.3 ***	16.5 ± 2.2 **	59.8 ± 5.7 ***
E 0.5 µM + P 5 50 µg/ml	11.2 ± 4.0 **	18.2 ± 4.5 ***	17.0 ± 1.0 **	53.5 ± 7.4 ***
E 0.5 µM + P 7 50 µg/ml	12.6 ± 3.0 ***	20.5 ± 3.7 ***	17.0 ± 2.7 **	50.0 ± 4.3 ***

The table shows mean results ± SD; *n* = 3; * *p* < 0.05, ** *p* < 0.01, *** *p* < 0.001.
